# Effectiveness of a digital therapeutic as adjunct to treatment with medication in pediatric ADHD

**DOI:** 10.1038/s41746-021-00429-0

**Published:** 2021-03-26

**Authors:** Scott H. Kollins, Ann Childress, Andrew C. Heusser, Jacqueline Lutz

**Affiliations:** 1 Department of Psychiatry and Behavioral Sciences, Duke Clinical Research Institute, Duke University, Durham, NC USA; 2 Center for Psychiatry and Behavioral Medicine, Las Vegas, NV USA; 3 Akili Interactive Laboratories, Boston, MA USA

**Keywords:** Paediatric research, Therapeutics

## Abstract

STARS-Adjunct was a multicenter, open-label effectiveness study of AKL-T01, an app and video-game-based treatment for inattention, as an adjunct to pharmacotherapy in 8–14-year-old children with attention-deficit/hyperactivity disorder (ADHD) on stimulant medication (*n* = 130) or not on any ADHD medication (*n* = 76). Children used AKL-T01 for 4 weeks, followed by a 4-week pause and another 4-week treatment. The primary outcome was change in ADHD-related impairment (Impairment Rating Scale (IRS)) after 4 weeks. Secondary outcomes included changes in IRS, ADHD Rating Scale (ADHD-RS). and Clinical Global Impressions Scale—Improvement (CGI-I) on days 28, 56, and 84. IRS significantly improved in both cohorts (On Stimulants: −0.7, *p* < 0.001; No Stimulants: −0.5, *p* < 0.001) after 4 weeks. IRS, ADHD-RS, and CGI-I remained stable during the pause and improved with a second treatment period. The treatment was well-tolerated with no serious adverse events. STARS-Adjunct extends AKL-T01’s body of evidence to a medication-treated pediatric ADHD population, and suggests additional treatment benefit.

## Introduction

Attention-deficit hyperactivity disorder (ADHD) is a neurodevelopmental disorder of persistent impaired attention, hyperactivity, and impulsivity that negatively affects daily functioning and quality of life. ADHD is one of the most commonly diagnosed pediatric mental health disorders, with a prevalence estimated to be 5% worldwide^1^ and exerts a substantial burden on families and society^2^. Attentional difficulties specifically are associated with impairment in several areas, such as academic functioning, shy and passive social behavior, and impaired adaptive functioning in children/adolescents^3^.

Front-line treatment for ADHD includes pharmacological and non-pharmacological interventions, which have demonstrated short-term efficacy^4–6^. However, there are limitations to both modalities of treatment^7–13^. Based in part on these limitations, there has been considerable interest in additional approaches to augment multimodal ADHD management. Digital therapeutics may offer improved access, minimal side effects, and low potential for abuse while providing targeted treatment options for improving cognitive functions, such as attention. A previous randomized controlled trial (STARS-ADHD), for example, demonstrated AKL-T01, a video-game based digital therapeutic, administered for 4 weeks at home, significantly improved attentional functioning as measured by the Test of Variables of Attention (TOVA^®^) in children diagnosed with ADHD, and a greater proportion of children on AKL-T01 showed evidence of improved ADHD-related impairment (IRS)^14^ compared to a digital control intervention after 4 weeks of treatment. Although the IRS change scores did not achieve statistical significance, a trend was observed (*p* = 0.09) that favored the active treatment.

However, this trial noted several important limitations, including eligibility criteria that only included children not taking ADHD medication and scoring below a specific objective attention functioning threshold on the TOVA at study entry. In addition, this study only evaluated treatment effects after a single 4-week treatment regimen.

The objective of the present trial, therefore, was to evaluate the AKL-T01 intervention as an adjunctive therapy in children currently taking stimulant medication for their ADHD, with no criteria pertaining to TOVA functioning at study entry. In addition, the present study evaluated a longer duration of intervention—specifically participants used AKL-T01 for 4 weeks (days 0–28), followed by a treatment break of 4 weeks (days 29–56) and a second treatment period of 4 weeks (days 57–84). By including a broader range of participants, another goal of the study was to evaluate the intervention under more “real-world” conditions and with a focus on clinical assessments related to ADHD-related impairment and symptoms. Specific to this point, we used the Impairment Rating Scale (IRS) as the primary outcome measure given findings from the previous study and its clinical relevance. It is important to note that this study was not designed to evaluate comparative efficacy and did not include a blinded control condition.

## Results

### Participants

**Fig. 1:**
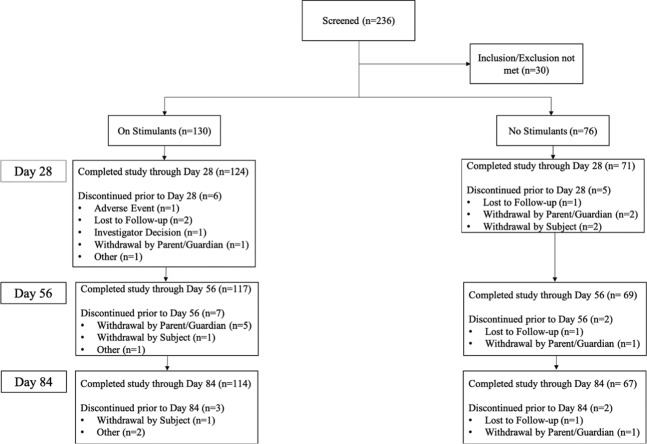
Trial Consort Table. Of the three AE-related withdrawals, one was coded as AE, two were coded as subject withdrawal (one in stimulant, one in no stimulant cohort).

A total of 236 participants were screened for inclusion in this study, with 206 meeting eligibility criteria (Fig. 1). One hundred and thirty were enrolled in the On Stimulants cohort, and 76 in the No Stimulants cohort. Baseline demographic characteristics of the cohorts are described in Table 1. Despite severe comorbidities being exclusionary, around 20% of the included participants still presented with at least one comorbidity according to DSM (for a full list of participant’s comorbidities see Supplementary Table 1). Both cohorts received AKL-T01 between December 2018 and September 2019. The study was registered on clinicaltrials.gov (NCT03649074).

**Table 1:** Demographics/baseline characteristics.

	On Stimulants cohort	No Stimulants cohort	Total
*N*	130	76	206
Age mean (SD)	10.6 (1.75)	10.5 (1.82)	10.6 (1.77)
Sex (male)	98/130 (75.4%)	56/76 (73.7%)	154/206 (74.8%)
Race			
White	106/130 (81.5%)	57/76 (75%)	163/206 (79.1%)
Black or African American	21/130 (16.2%)	16/76 (21.1%)	37/206 (18.0%)
American Indian or Alaska Native	3/130 (2.3%)	1/76 (1.3%)	4/206 (1.9%)
Asian	8/130 (6.2%)	2/76 (2.6%)	10/206 (4.9%)
Native Hawaiian or Other Pacific Islander	0	1/76 (1.3%)	1/206 (0.5%)
Other	0	4/76 (5.3%)	4/206 (1.9%)
Ethnicity			
Hispanic or Latino	24/130 (18.5%)	20/76 (26.3%)	44/206 (21.4%)
Not Hispanic or Latino	106/130 (81.5%)	54/76 (71.1%)	160/206 (77.7%)

Overall, 195/206 (95%) participants completed the study through the primary endpoint at the end of the initial 4-week AKL-T01 treatment period (Day 28); 186/206 (90%) completed through 8 weeks (Day 56: 4 weeks of treatment, 4 weeks treatment pause); and 179/206 (87%) completed all 12 weeks of the study (Day 84: 4 weeks of treatment, 4 weeks treatment pause, additional 4 weeks of treatment). The On Stimulants cohort remained on stimulants during the AKL-T01 treatment pause.

In general, participants remained consistent with respect to their assigned treatment group throughout the trial. All children in the No Stimulants cohort remained off stimulants during the first AKL-T01 treatment period and the treatment pause period. Several participants in the On Stimulants cohort stopped their stimulant medication (two children during the first AKL-T01 treatment period, and two during the AKL-T01 treatment pause). Information on crossover is not available in the 2nd treatment period.

### Compliance

All participants were instructed to play AKL-T01 for ~25 min/day, 5 days/week, for each 4-week treatment period. Within each day, the 25-min sessions were divided into five 5-min “missions.” As such, participants were recommended to play up to 100 missions over the course of a 4-week treatment (5 missions/day × 5 days/week × 4 weeks). In the first treatment month, the mean (SD) number of missions played in the intention-to-treat (ITT) population was 81.1 (28.37) missions for the On Stimulants cohort and 73.0 (33.83) missions for the No Stimulants cohort. Across the full study and 2 treatment months, the overall mean (SD) number of missions played in the ITT population was 135.2 (56.20) missions for the On Stimulants cohort and 116.4 (65.69) sessions for the No Stimulants cohort (of 200 recommended missions). In the previously published RCT, children were required to play 50% or more of the recommended missions as part of the per protocol population^14^. In the present study, therefore, the 50% compliance criterion was applied during the first treatment month as an additional compliance indicator. For comparison in the present study, 79.1% of children met this criterion versus 85.6% in the previous RCT.

Supplementary Figure 1 provides a more granular view of compliance over time, showing the percent of children who used AKL-T01 at least once during a treatment week, as well as the average of missions played across all children in a given week. Note that study dropout data are only available per clinic visit and not by week, and are available in the Consort table and Supplementary Fig. 1. Exploratory tabulations of baseline demographics for children who played less than or more than 50% of the recommended missions and the children who dropped out are given in the Supplementary Table 2.

### Efficacy outcomes

**Fig. 2:**
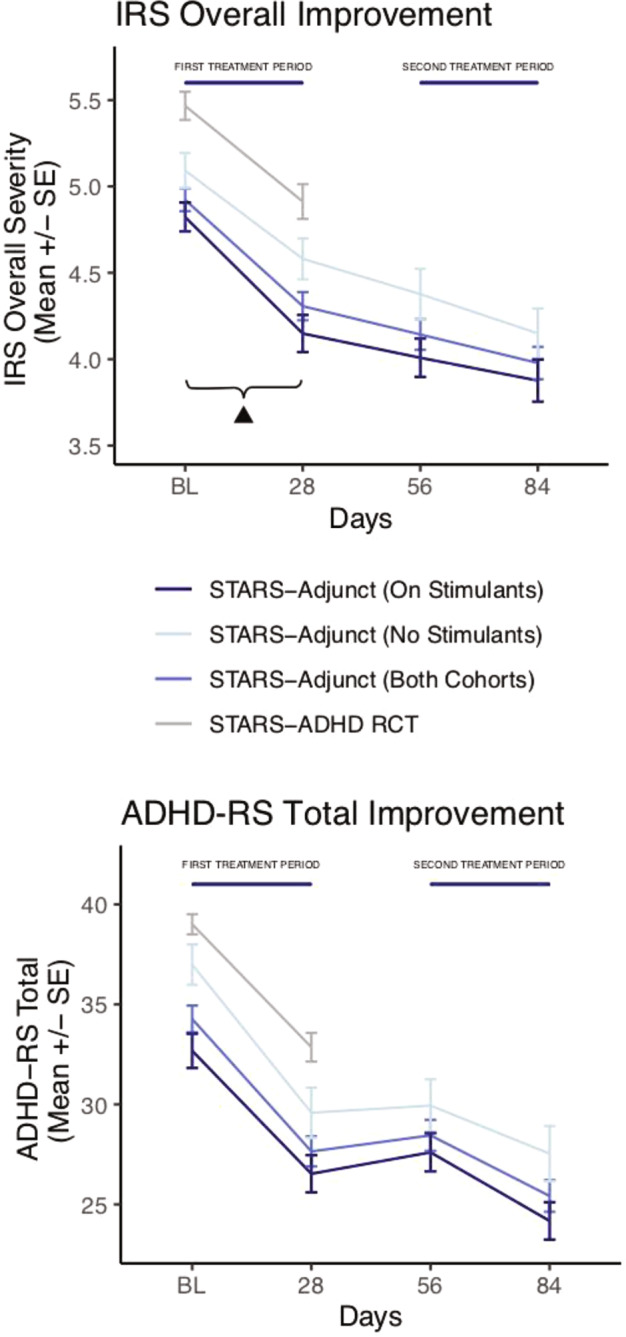
Clinical measures of ADHD symptoms (ADHD-RS) and ADHD-related impairment (IRS) improve with AKL treatment and remain stable during a 4-week treatment pause. ▴Primary outcome in the STARS-Adjunct trial was the change in IRS from baseline (BL) to Day 28 in the two cohorts. Data distribution for each group and time point is displayed in Supplementary Fig. 1.

AKL-T01 significantly improved (lowered) ADHD-related impairment as measured with the IRS (clinician-rated) after the first 4-week treatment in both cohorts (primary endpoint): with mean changes from Baseline to Day 28 in IRS overall severity score of −0.7 (95% confidence interval (CI): [−0.86, −0.50]; DOF: 127; Cohen’s d: .65; *p* < 0.001) in the On Stimulants cohort and −0.5 (95% CI: [−0.73, −0.32]; DOF: 73; Cohen’s d: .59; *p* < 0.001) in the No Stimulants cohort (Fig. 2). Secondary clinical endpoints were changes in ADHD symptoms (clinician-rated ADHD-RS Total and ADHD-RS Inattention and Hyperactivity-Impulsivity subscale score mean changes from Baseline to Day 28) and the mean clinical global impairment (CGI-I score at Day 28 compared to a score of 4, i.e., no change). All secondary clinical measures showed improvement in both cohorts (all *p* < 0.001) (Table 2).

**Table 2:** STARS-Adjunct Secondary Endpoints (ITT), change from Baseline to Day 28.

	STARS-Adjunct								
	On Stimulants (*N* = 130)			No Stimulants (*N* = 76)			Both cohorts (*N* = 206)		
	Baseline Mean (SD) *n*	Change Mean (SD) *n*	(95% CI) Cohen’s d t-statistic *p* value	Baseline Mean (SD) *n*	Change Mean (SD) *n*	(95% CI) Cohen’s d t-statistic *p* value	Baseline Mean (SD) *n*	Change Mean (SD) *n*	(95% CI) Cohen’s d t-statistic *p* value
ADHD-RS Total	32.7 (9.8) 130	−6.1 (7.18) 128	(−7.36, −4.85) 0.85 −9.61 <0.001	37.0 (8.78) 76	−7.4 (9.92) 74	(−9.66, −5.07) 0.74 −6.39 <0.001	34.27 (9.64) 206	−6.56 (8.29) 202	(−7.71, −5.42) 0.79 −11.26 <0.001
ADHD-RS Inattention subscale	18.7 (5.18) 130	−3.4 (4.43) 128	(−4.17, −2.62) 0.77 −8.66 <0.001	21.1 (3.98) 76	−3.9 (5.60) 74	(−5.24, −2.65) 0.70 −6.06 <0.001	19.6 (4.9) 206	−3.59 (4.89) 202	(−4.27, −2.92) 0.74 −10.45 <0.001
ADHD-RS Hyperactivity-Impulsivity subscale	14.0 (6.58) 130	−2.7 (3.92) 128	(−3.40, −2.03) 0.69 −7.83 <0.001	15.9 (6.35) 76	−3.4 (5.13) 74	(−4.61, −2.23) 0.67 −5.74 <0.001	14.67 (6.54) 206	−2.97 (4.4) 202	(−3.58, −2.36) 0.68 −9.60 <0.001
CGI-I	–	3.3 (0.84) 128	(3.17, 3.47) 0.81 −9.14 <0.001	–	3.4 (0.83) 74	(3.23, 3.61) 0.70 −6.04 <0.001	–	3.36 (0.84) 202	(−0.76, −0.53) 0.77 −10.95 <0.001

Degrees of freedom for two-sided within-group *t*-tests on change scores are equal to *n* − 1.

To evaluate the stability of effects after a 4-week treatment pause and the effects of an additional treatment period, we conducted exploratory analyses of our endpoints for Day 56 (after the treatment pause) and Day 84 (after the second 4-week AKL-T01 treatment). Mean changes from baseline to Day 56 in IRS overall severity score, ADHD-RS total score, and Inattention and Hyperactivity-Impulsivity subscale scores remained significantly improved for participants in both cohorts (all *p* < 0.001). These treatment effects increased to Day 84 from baseline in both cohorts (Fig. 2). Similarly, the CGI-I score continued to be significantly below 4 (“no change”) at Day 56 in both cohorts (all *p* < 0.001) and further slightly improved at Day 84 in both cohorts.

### Responder results

A majority of participants (55.5%) in the On Stimulants cohort were classified as responders on the primary outcome of the IRS (improvement of ≥1 point on the IRS total score from Baseline to Day 28). In the No Stimulants cohort, 40.5% of participants were IRS responders. ADHD-RS symptom response rates were similar for children on and off ADHD medication with over 40% of responders across both cohorts after the second 4-week treatment. Table 3 illustrates the percentages of responders for each cohort after the first month (Day 28, a priori defined) across both cohorts, and after the second treatment month (Day 84, post hoc).

**Table 3:** Responder Analyses.

Outcome measure	Response rate definition	AKL-T01 response rate: STARS-Adjunct					
		No Stimulants		On Stimulants		Both cohorts	
		Day 28 (*n*/*N*) %	Day 84 (*n*/*N*) %	Day 28 (*n*/*N*) %	Day 84 (*n*/*N*) %	Day 28 (*n*/*N*) %	Day 84 (*n*/*N*) %
IRS overall responder	% children with ≥1 point improvement on IRS Overall Score	30/74 40.5%	46/67 68.7%	71/128 55.5%	77/113 68.1%	50%	68.3%
ADHD total symptoms (ADHD-RS Total)	% children with ≥30% improvement on ADHD-RS Total	20/74 27%	33/67 49.3%	35/128 27.3%	49/114 43%	27.2%	45.3%
CGI-I	% children with score of ≤2 on CGI-I	10/74 13.5%	17/67 25.4%	20/128 15.6%	33/114 28.9%	14.9%	27.6%

In post hoc analyses, the response rate for children On Stimulants was higher in the IRS at Day 28 (after 4 weeks) (*p* = 0.041) but this difference was not found at Day 84 (*p* = 0.94). None of the other responder comparisons between groups were significant.

### Parent/patient perspective

A majority of parents and children indicated a perceived improvement in ability to pay attention after the trial. Proportions in the study were comparable to the previous STARS-ADHD study (Table 4).

**Table 4:** Parent and child perspective: improved attention.

	Study/day	Description	Both cohorts
Parent Perspective Improved Attention	STARS-Adjunct/Day 84	% parents/children who answered “Yes” to question: do you think playing the app improved your child’s (your) ability to pay attention	60%
	STARS-ADHD/Day 28		56%
Child Perspective Improved Attention	STARS-Adjunct/Day 84		75%
	STARS-ADHD/Day 28		73%

### Safety results

Overall, 37 (18%) participants experienced a device-related adverse event (AE) during the 12-week trial. AEs were generally evenly distributed between the On Stimulants and No Stimulants cohorts. The most common device-related AEs were decreased frustration tolerance, headache, and irritability (Table 5). All device-related AEs were either mild or moderate in severity, with no serious AEs reported. Three participants discontinued treatment due to a treatment-related AE (all decreased frustration tolerance).

**Table 5:** Device-related adverse events by system organ class and preferred term (safety population).

System organ class	System organ subclass	On Stimulants	No Stimulants	Total
Preferred term		*N* = 130	*N* = 76	*N* = 206
Any device-related adverse event	Total	22/130 (16.9%)	15/76 (19.7%)	37/206 (18.0%)
Psychiatric	Total	17/130 (13.1%)	12/76 (15.8%)	29/206 (14.1%)
	Frustration tolerance decreased	15/130 (11.5%)	12/76 (15.8%)	27/206 (13.1%)
	Irritability	2/130 (1.5%)	1/76 (1.3%)	3/206 (1.5%)
	Agitation	1/130 (0.8%)	0/76 (0.0%)	1/206 (0.5%)
	Anxiety	0/130 (0.0%)	1/76 (1.3%)	1/206 (0.5%)
Nervous system	Total	4/130 (3.1%)	2/76 (2.6%)	6/206 (2.9%)
	Headache	3/130 (2.3%)	1/76 (1.3%)	4/206 (1.9%)
	Dizziness	1/130 (0.8%)	1/76 (1.3%)	2/206 (1.0%)
Eye	Total	1/130 (0.8%)	0/76 (0.0%)	1/206 (0.5%)
	Asthenopia	1/130 (0.8%)	0/76 (0.0%)	1/206 (0.5%)
Gastrointestinal	Total	1/130 (0.8%)	0/76 (0.0%)	1/206 (0.5%)
	Nausea	1/130 (0.8%)	0/76 (0.0%)	1/206 (0.5%)
General	Total	1/130 (0.8%)	0/76 (0.0%)	1/206 (0.5%)
	Feeling abnormal	1/130 (0.8%)	0/76 (0.0%)	1/206 (0.5%)
Skin and subcutaneous tissue	Total	0/130 (0.0%)	1/76 (1.3%)	1/206 (0.5%)
	Pruritus	0/130 (0.0%)	1/76 (1.3%)	1/206 (0.5%)

All enrolled participants who received a device. Participant’s cohort was determined based on where the participant spent the majority of the first treatment phase. The denominator for the percentages is the number of participants in each cohort and all. At each level of summarization (System Organ Class or Preferred Term), participants who experienced more than one adverse event were counted only once. All adverse events were coded using the Medical Dictionary for Regulatory Activities (MEDRA) version 22.0.

## Discussion

The present study adds to and extends the clinical evidence base for AKL-T01, a video-game based treatment for improving attentional functioning in children with ADHD. Results address limitations from previous studies in several ways. First, children who were concurrently treated with stimulant medication showed comparable benefits on impairment and ADHD symptoms compared to children not taking stimulant medication. Second, results show that effects persist during a 4-week treatment pause and further improve with a second 4-week treatment period. Finally, this study demonstrates improvement in clinically relevant (symptoms and impairment) outcomes for children without pre-existing attentional impairment, as measured by the TOVA in previous studies. Collectively, these results increase the generalizability of previous studies and provide further evidence that AKL-T01 is a viable option for improving attention functioning in children with ADHD, including children receiving concurrent pharmacotherapy.

A previously published randomized controlled trial of AKL-01 (STARS-ADHD) reported significant improvements in attentional functioning (TOVA API) and evidence for improvement in ADHD-related impairment (IRS) after 28 days of treatment compared to a control group who received a tablet-based digital control word game^14^. This previous study required children who were taking medication to discontinue use prior to randomization or to be naive to medication. Given that the majority of children diagnosed with ADHD take medication to help manage their condition^12^, it is important to evaluate the effects of new non-pharmacological interventions in the context of routine care. Moreover, based on existing literature^15^, practice guidelines typically recommend a multimodal approach for treating ADHD that includes both medication and behavior therapy^16^. Few studies have examined the effects of combining pharmacotherapy with other kinds of non-pharmacological interventions in children with ADHD. The few trials that studied cognitive training approaches as adjunctive to ADHD medication in children with ADHD show inconclusive results regarding additional benefits of such interventions in children with ADHD medication^17,18^. Results from the present study showed that the effects of AKL-T01 on ADHD-related impairment and ADHD symptoms were generally similar in children taking stimulant medication versus those not taking any medication.

The previous trial also excluded children whose objectively measured attentional functioning on the TOVA was not significantly impaired (i.e., API score < −1.8). This resulted in a number of children who otherwise met criteria for ADHD not being eligible. In the present study, there were no eligibility criteria for enrollment based on attentional functioning. By including both children who were taking medication and whose objectively measured attentional functioning was free to vary, the sample was more representative of the general population of pediatric patients with ADHD.

The duration of treatment (4 weeks) in the original study^14^ was brief and the effects of continued, additional treatment periods with AKL-T01 were not explored. The current study addressed this by examining two separate 4-week bouts of AKL-T01 treatment with a treatment pause of 4 weeks in between the treatment periods. Results showed that outcome measures remained stable during the treatment pause and continued to improve following the second treatment period, and these patterns were seen in children on and off stimulant medication. These effects are similar to those observed in other open-label studies of both non-pharmacological (i.e., behavior therapy) and pharmacological treatments^19–22^. In general, the magnitude of changes in ADHD symptoms after treatment were comparable to other non-pharmacological interventions. For example, a randomized, blinded, sham-controlled trial of trigeminal nerve stimulation—a treatment that has received FDA clearance for ADHD—reported effect sizes for ADHD-RS scores of approximately *d* = 0.5, compared to an effect size of *d* = 0.79 in the current, unblinded study^23^. Additional research is needed to better contextualize the comparative efficacy of AKL-T01 compared to other non-pharmacological treatment approaches for managing ADHD symptoms.

Results of the present study should be interpreted in light of several important limitations. First, the study was conducted without randomization or a blinded control group, thus making it impossible to rule out the possibility of placebo effects accounting for the findings or regression to the mean effects. But we observed similar outcomes in the IRS and ADHD-RS, with a similar magnitude of treatment effects for both the On Stimulants and No Stimulants cohorts, which was also comparable to those observed in the previously published STARS-ADHD RCT. This comparability suggests consistency in the degree to which AKL-T01 is associated with changes in parent and clinician-reported impairment and ADHD symptoms. In addition, the generally larger improvements in all measures during treatment, compared to the treatment pause suggest that effects were not merely regression to the mean. Moreover, given that one of the study aims was to examine longer-term treatment outcomes, maintaining participants on a control intervention for 3 months would have been challenging. Nevertheless, it is important to not interpret the present findings as evidence of efficacy in the absence of a control group, but rather as a demonstration that previously reported results are consistent in a sample more closely resembling that which would be seen in clinical practice, allowing a more clinically meaningful view on extended treatment regimens with AKL-T01.

Second, the present study still excluded children with significant comorbidity. Although children with some conditions such as ODD and mild anxiety were allowed to participate (see Supplementary Table 1) provided these conditions did not interfere with participation and that ADHD was still the primary diagnosis, the full generalizability of AKL-T01 was not investigated in this study. It is well established that comorbidity is common in children (as well as adolescents and adults) with ADHD, and future research on the effects of AKL-T01 in such populations will be important to help guide clinical practice.

Third, the present study, while increasing the duration of treatment exposure to 2 months, and total study duration to 3 months, does not provide information on the long-term effects of AKL-T01 on ADHD-related impairment and functioning. By definition, ADHD is a chronic condition and understanding long-term treatment effects is critically important—and relatively understudied even with conventional treatment modalities^24,25^. The present study provides some evidence that 4-week treatment leads to stable effects for at least 4 weeks and continued treatment leads to continued improvement for some outcomes. This is plausible given the hypothesized mechanism of action of AKL-T01 and previous mechanistic studies that have shown long-term improvements in symptoms^26^ and cognition with AKL-T01 in children with SPD and improvements in cognition in healthy older adults^27^. It will be important to continue to study the effects of repeated exposures to the treatment to better inform “dosing” of the treatment in the real world.

Future trials are necessary and underway to further establish performance of the intervention in the context of integrated clinical ADHD treatment settings, and evidence generation for digital therapeutics such as AKL-T01 should also build on RCTs^28,29^. Qualitative human-centered investigations and pragmatic RCT approaches have been used in health services research for digital health-enabled clinical service implementation—for example, to assess solutions for depression, anxiety, pain, and diabetes^30–32^. These are promising avenues for research of the AKL-T01 digital therapeutic. Such studies can also help establish clinical guidance around which children would benefit the most from the intervention.

These limitations notwithstanding, the present study adds to the growing evidence base for the potential benefits of AKL-T01 in pediatric ADHD. The continued examination of the extent of treatment effects, as well as the generalizability and durability of effects will be important. As noted, continued evaluation of the effects of AKL-T01 on other important aspects of functioning like academic and social functioning, health utilization and health outcomes would continue to add to the evidence base that the effects observed in this and previous studies have substantial clinical and functional impact.

## Methods

### Overview

STARS-Adjunct (NCT03649074) was a multisite, open-label study in children/adolescent 8–14 years old with a confirmed diagnosis of ADHD, primarily inattentive or combined subtype (per DSM-V and MINI-KID), experiencing suboptimal treatment response (IRS ≥ 3 Overall Impairment Score), at Screening. Participants had to be either stable on stimulant medication (On Stimulants cohort) or off any ADHD medication (No Stimulants cohort), and not present other significant comorbid psychiatric diagnoses. All participants had an IQ score ≥80 (per KBIT-II). The study was conducted in accordance with the International Conference on Harmonisation Regulations, and was approved by each site’s institutional review board (Copernicus Group/WIRB [11 sites], Cincinnati Children’s Hospital Medical Center, UC Davis, USCF, Johns Hopkins Medical Center [1 site each]. The overall study IRB was Duke University Health System). All participants and their caregivers provided written or verbal assent and written consent, respectively, prior to any study activities being conducted.

### Study design

The study planned to enroll 203 participants with a confirmed diagnosis of ADHD at 15 sites. Participants were divided between two cohorts: 130 participants were planned to enroll in the On Stimulants cohort, and 73 participants were planned to enroll in the No Stimulants cohort.

**Fig. 3:**
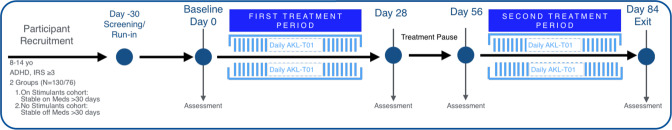
STARS-Adjunct study overview. The figure shows a schematic of the design of the current study.

During the first 4-week AKL-T01 treatment phase (Days 1 through 28) participants in the On Stimulants cohort received stimulants plus AKL-T01, and participants in the No Stimulants cohort received AKL-T01 only. During the 4 weeks AKL-T01 treatment pause (Days 29 through 56) between treatment phases, participants in the On Stimulants cohort remained on stimulant, and participants in No Stimulants cohort remained off ADHD medication. During the second 4-week AKL-T01 treatment phase (Days 57 through 84), participants in the On Stimulants cohort received stimulants plus AKL‑T01, and participants in the No Stimulants cohort received AKL‑T01 (Fig. 3).

### Participants

Eligible patients were male/female children/adolescents aged 8–14 years old with a confirmed diagnosis of ADHD (as per the Diagnostic and Statistical Manual of Mental Disorders (5th edn)), primarily inattentive or combined subtype (per DSM-V and MINI-KID), experiencing suboptimal treatment response, as reflected by ADHD-related impairment (IRS Overall Impairment Score ≥3, parent-rated) at screening, and no significant comorbid psychiatric diagnoses that would make participation in the study difficult. All participants had an IQ score ≥80 (per KBIT-II). For the On Stimulants cohort, participants had to be on a stable dose of stimulant medication, at an approved dose, for at least 30 days prior to enrollment and show a moderate response on a stimulant, with room for improvement. For the No Stimulants cohort, participants had to be stable without a stimulant or any other ADHD medication for at least 30 days before the baseline visit. Complete inclusion and exclusion criteria are in Supplementary Note 1.

### Procedures

The AKL-T01 intervention was preloaded and administered on iPad mini 2 tablets (Apple, USA). At the baseline visit, eligible patients were instructed to use AKL-T01 for about 10 min while a study coordinator monitored the session to ensure that patients could follow the rules of AKL-T01. Patients were further assessed on ADHD-related impairment (IRS) and symptoms, and attentional functioning at the baseline visit.

Children then played the AKL-T01 digital treatment at home, for 4 weeks (Days 1–28), followed by a 4-week treatment pause (Days 29–56) and then a second 4-week AKL-T01 treatment (Days 57–84). Study visits were scheduled on Days 28, 56, and 84. During the AKL-T01 intervention periods, patients were instructed to use AKL-T01 at home for 5 days per week, for 4 weeks. A daily session consisted of five 4–5-min multitasking “missions” (total time on task was ~25 min). Compliance was monitored electronically. AKL-T01 generated automatic reminders.

AKL-T01 is a digital therapeutic that uses a proprietary algorithm designed to improve attentional control, by training interference management (multitasking). AKL-T01 mechanisms have been described previously^14,27,33^. In brief: users multitask by responding to a perceptual discrimination targeting task and a simultaneous sensory motor navigation task. Users advance by reducing interference costs (closes the performance gap between multitasking and single-tasking), and real-time and periodic recalibration occurs to maintain an optimal difficulty level.

### Outcomes

The primary endpoint was the change in ADHD-related impairment as measured by the (parent-reported, clinician-rated) from Baseline to Day 28. ADHD-related impairment is assessed across domains such as social functioning, academic progress, and self-esteem, including an overall impairment severity score.

Secondary endpoints included the overall change in ADHD-RS Total Score from Baseline to Day 28 (parent-reported clinician-administered assessment of the child’s frequency of ADHD symptoms), the Clinical Global Impressions (CGI) is a clinician-rated assessment of a patient’s global functioning, scored from 1—very much improved to 7—very much worse, with 4 being no improvement. Secondary endpoints related to attention functioning (TOVA) were assessed but will be reported separately.

Exploratory outcomes included clinical measures related to ADHD symptoms and impairment after the 4-week AKL-T01 treatment pause (Day 56) and after the second 4-week AKL-treatment phase (Day 84).

### Statistical analysis

All analyses were performed according to a prespecified statistical analysis plan, unless otherwise specified as post hoc. Unless otherwise indicated, statistical comparisons used a two-sided significance test evaluated at the 95% level of confidence.

Unless otherwise specified, all outcomes were analyzed using an ITT methodology (defined as any participant enrolled in the study who received a device).

Sample size estimation was performed and determined that an effective total of at least 66 non-medicated participants (No Stimulants cohort) would be needed to demonstrate an effect size of 0.40 with 90% power and 95% confidence. This target effect size was determined from our previous RCT study (STARS-ADHD), where the within-group effect size on the primary (IRS) was 0.38. Since there was no prior data to estimate an effect size for the medicated participants (On Stimulants cohort), we hypothesized that we might observe an attenuated effect (~25% decrease in effect size) and so this cohort was powered for a 0.30 effect size. This analysis revealed that at least 117 medicated participants (cohort 1) would be needed to demonstrate an effect size of 0.30 with 90% power at the 95% confidence level using a within-group *t*-test. Incorporating a 10% dropout rate required that at least 130 + 73 = 203 participants were planned to be enrolled in the study.

All analyses were conducted using a complete case analysis. In no situation were missing data to be imputed.

Statistical methodology for the primary, secondary, and exploratory endpoints are summarized below. For a complete list, please see Supplementary Note 2.

Within each cohort, the primary efficacy endpoint for each participant was the change in the clinician-reported IRS “overall severity of child’s problem in functioning and overall need for treatment” score from baseline to Day 28, defined as the score on Day 28 minus the score at baseline. Missing data were not imputed. If a participant was missing either the baseline or Day 28 IRS assessment, that participant was excluded from the analysis. The results of the primary efficacy analysis were summarized by cohort as mean change from baseline (95% CI). Significance was assessed with a two-sided paired *t*-test evaluated at the 95% level of confidence.

The following secondary efficacy endpoints were tested using the same technique outlined for the primary efficacy analysis: (1) change in ADHD-RS from baseline to Day 28, (2) CGI-I score at Day 28, and (3) change in TOVA constituents from baseline to Day 28. TOVA results will be reported elsewhere.

Family-wise error rate was controlled using a hierarchical testing strategy. Within each cohort, the maximum allowable Type I error rate was set to 0.05. For additional details, please see Supplementary Note 3.

Exploratory analyses included the second treatment month (Day 84) and interim pause time point (Day 56) for the primary and secondary endpoints (described in detail in Supplementary Note 3).

Planned responder analyses were conducted for 1 point improvement on IRS “overall severity of child’s problem in functioning and overall need for treatment” score (change from Day 0 to Day 28), 30% improvement on ADHD-RS total score at Day 28 and score of 2 or better on the CGI-I at Day 28. Post hoc response rates were calculated for Day 0 to Day 84 (after the second 4-week AKL-T01 treatment).

This was a multicenter study. The primary endpoint was assessed for poolability across sites within each cohort using a linear mixed model where site was a random effect (there was no significant effect of site and cohort (*p* = 0.152)).

### Reporting summary

Further information on research design is available in the Nature Research Reporting Summary linked to this article.

## Supplementary information

Supplementary Information

Reporting Summary

## Data Availability

The STARS-Adjunct Investigators agree to share de-identified individual participant data, the study protocol, and the statistical analysis plan with academic researchers 6 months after publication, and following completion of a Data Use Agreement. Proposals should be directed to medinfo@akiliinteractive.com.
