# Supplementary material for: Effectiveness of a digital therapeutic as adjunct to treatment with medication in pediatric ADHD
Source: NPJ Digit Med. 2021 Mar 26;4:58. doi: 10.1038/s41746-021-00429-0 (PMC7997870; doi:10.1038/s41746-021-00429-0)
Supplement: Supplementary file 1 — Supplementary Information [file 41746_2021_429_MOESM1_ESM.pdf]

Supplementary Material

Supplementary Note 1: Full inclusion/exclusion criteria:

Inclusion Criteria:

- Male and female children between the ages of 8-14 years
- Confirmed ADHD diagnosis (primarily inattentive or combined subtype) at Screening based on DSM-V criteria and established via the MINI-KID, administered by a trained clinician.
- Experiencing suboptimal treatment of ADHD (IRS ≥ 3 Overall Impairment Score), at Screening.
- Estimated IQ score ≥ 80 as assessed by the Kaufmann Brief Intelligence Test, Second Edition (KBIT-II).
- For the On Stimulants cohort, participants must have been stable on stimulant medication, at an approved dose, for ≥ 30 days prior to enrollment.
- For the No Stimulants cohort, participants must be stable off stimulant medication for ≥ 30 days prior to enrollment.

Exclusion Criteria:

- Current, controlled (requiring a restricted medication) or uncontrolled, comorbid psychiatric diagnosis, based on MINI-KID
- Participants currently treated with a nonstimulant medication for ADHD (i.e., atomoxetine, clonidine, or guanfacine).
- Initiation or planned change of behavioral therapy within the last 4 weeks from the time of consent or during trial.
- Participant was considered a suicide risk in the opinion of the Investigator, had previously made a suicide attempt, or had a prior history of, or was demonstrating active suicidal ideation or self-injurious behavior as measured by Columbia-Suicide Severity Rating Scale (C-SSRS) at Screening.
- Motor condition (e.g., physical deformity of the hands/arms; prostheses) that prevented playing the digital treatment as reported by the parent or observed by the investigator.
- Recent history (within the past six months) of suspected substance abuse or dependence.
- History of seizures (exclusive of febrile seizures), or significant motor or vocal tics, including but not limited to Tourette's Disorder.
- Diagnosis of or parent-reported color blindness (Confirmed in-clinic via Ishihara Color Blindness Test).
- Uncorrected visual acuity (confirmed via ability of participant to log-in to T01 app and complete in-clinic game play, at baseline).
- Regular use of psychoactive drugs (nonstimulant) that in the opinion of the Investigator may confound study data/assessments.

Supplementary Note 2: Endpoints

Primary (In Cohort 1, Cohort 2):

Overall change in clinician-reported IRS "Overall severity of child's problem in functioning and overall need for treatment" score from baseline to Day 28.

Secondary (In Cohort 1, Cohort 2):

1. Overall change in ADHD-RS total component score from baseline to Day 28.
2. Clinical Global Impression-Improvement (CGI-I) score at Day 28
3. Overall change in TOVA attention composite score (ACS) and certain constituent scores (Ex-Gaussian Tau Total, Commission Errors Standard Score H2, RT Variability Standard Score Total, RT Mean Standard Score H1, D-Prime Standard Score H2, and Omission Errors Standard Score H2), from baseline to Day 28.

Exploratory (In Cohort 1, Cohort 2):

1. Overall change in clinician-reported IRS "Overall severity of child's problem in functioning and overall need for treatment" score from baseline to Day 56 and from baseline to Day 84.
2. Overall change in ADHD-RS total component score from baseline to Day 56 and from baseline to Day 84.
3. CGI-I score at Day 56 and at Day 84.
4. Overall improvement in TOVA ACS and certain constituent scores (described in secondary section), from baseline to Day 56 and from baseline to Day 84.
5. Overall change in Test of Silent Reading Efficiency and Comprehension (ToSREC) from baseline to Day 28 and from baseline to Day 84, using the appropriate ToSREC form.

Supplementary Note 3: Methods

Secondary Efficacy Endpoints Hierarchical Testing Strategy Details

Family-wise error rate was controlled using a hierarchical testing strategy. Within each cohort, the maximum allowable Type I error rate was set to 0.05. If the primary efficacy endpoint was significant at the 0.05 confidence level, the secondary efficacy endpoints were tested sequentially in the order in which they appear above, starting with (1) change in ADHD-RS Inattention sub-scale. If the test for (1) was significant at the 0.05 confidence level, the test for (2) change in autocalculated sum of ADHD-RS Total was to proceed. If the test for (2) was significant at the 0.05 confidence level, the test for (3) CGI-I score at Day 28 was to proceed. If the test for (3) was significant at the 0.05 confidence level, the test for TOVA constituents from baseline to Day 28 was to proceed, starting with API/ACS, and then to Ex-Gaussian Tau Total, Commission Errors Standard Score H2, RT Variability Standard Score Total, RT Mean Standard Score H1, D-Prime Standard Score H2, and Omission Errors Standard Score H2, in this order. If significant at the 0.05 significance level, the test for change in ADHD-RS Hyperactive subscale was to proceed. At any point, if the test of a secondary efficacy endpoint failed to achieve significance at the 0.05 confidence level, only nominal p-values were reported for that endpoint and for all subsequent secondary efficacy endpoints. If the primary efficacy endpoint was not significant at the 0.05 confidence level, only nominal p-values were to be reported for all secondary efficacy endpoints

Supplementary Figure 1: Additional Compliance metrics and figures

Figure SA.4

a)

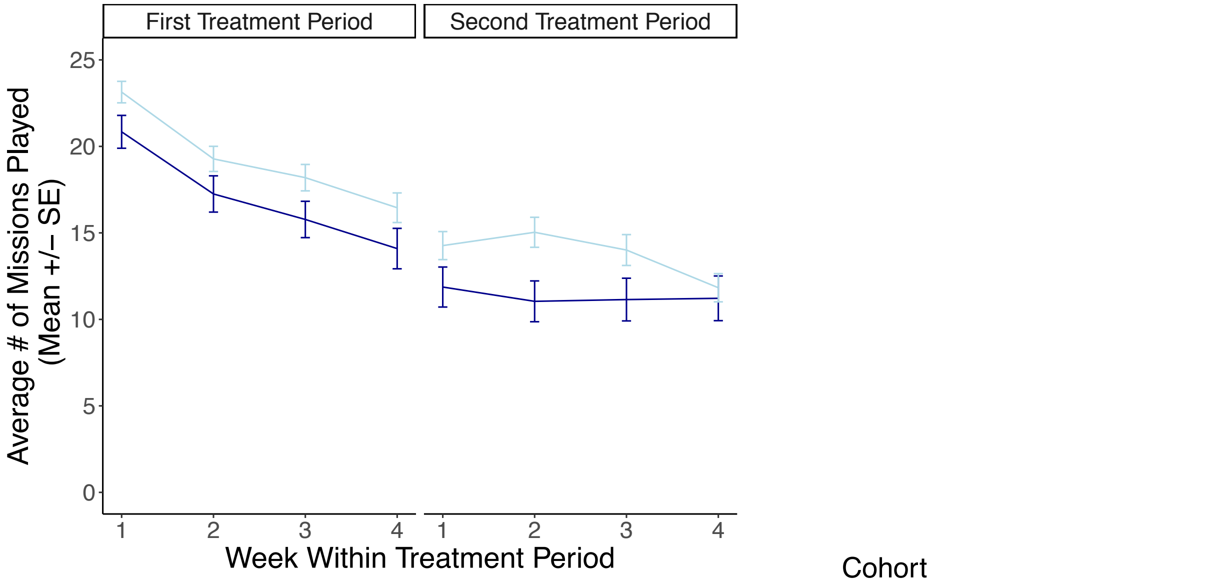

b)

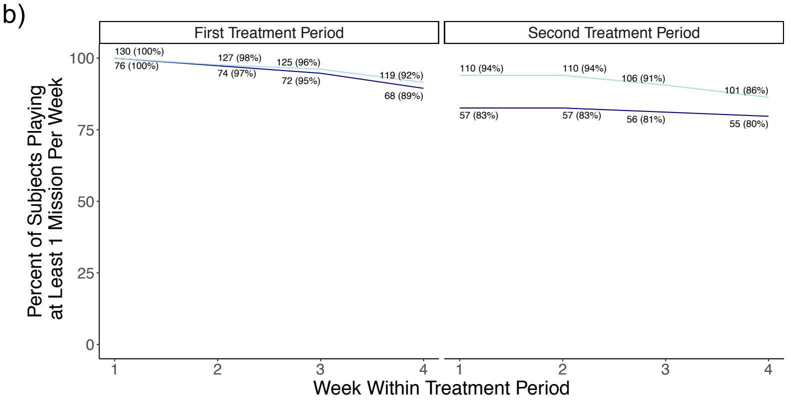

c)

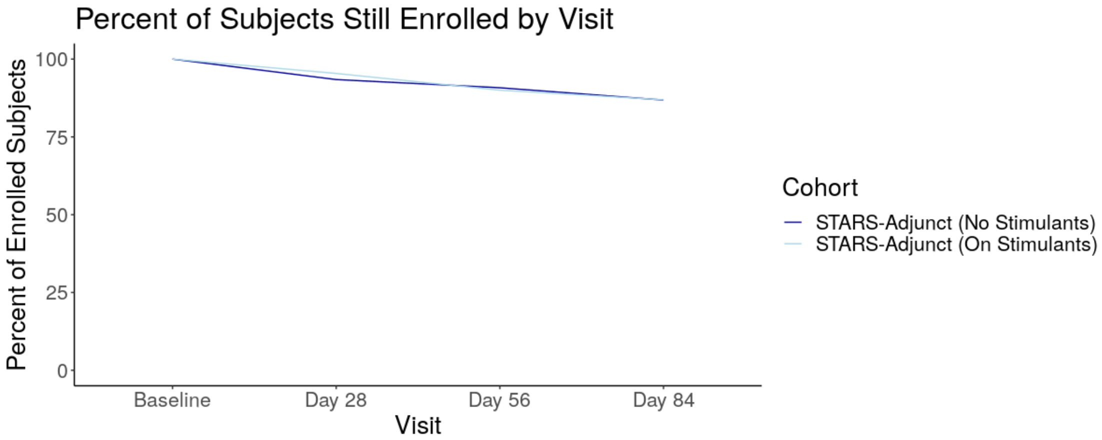

Visual representations of weekly usage and compliance measures during the two AKL-T01 treatment periods showing the a) average missions played (Basis: all children in the study in a given month even if they did not play in a given week) and the b) percentage of children playing at least once in a given week (Basis: all children in the study in a given month). Graph c) shows the percentage of remaining study participants per month in % of total enrolled participants by study month

Supplementary Table 1: Participants with comorbid disorders (DSM-5)

**Participants without any comorbidity**		n	%
Overall		163	79%
	On Stimulants Cohort	101	78%
	No Stimulants Cohort	62	82%

**Participants with at least 1 comorbidity**		n	%
Overall		43	21%
	On Stimulants Cohort	29	22%
	No Stimulants Cohort	14	18%

Listing of specific comorbidities (DSM-5)

On Stimulants Cohort

SubID	Comorbidity	
201001	Disruptive, Impulse-Control, and Conduct Disorders	Oppositional Defiant Disorder
201007	Sleep-Wake Disorders	Insomnia Disorder (intermittent)
201009	Disruptive, Impulse-Control, and Conduct Disorders	Oppositional Defiant Disorder
201011	Sleep-Wake Disorders	Insomnia Disorder
201012	Sleep-Wake Disorders	Insomnia Disorder
220004	Elimination Disorders	Enuresis
218001	Neurodevelopmental Disorders	Specific Learning Disorder
218001	Anxiety Disorders	Generalized Anxiety Disorder
218001	Disruptive, Impulse-Control, and Conduct Disorders	Oppositional Defiant Disorder
218001	Anxiety Disorders	Social Anxiety Disorder (Social Phobia)
216004	Neurodevelopmental Disorders	Specific Learning Disorder
220007	Sleep-Wake Disorders	Insomnia Disorder
220010	Anxiety Disorders	Social Anxiety Disorder (Social Phobia)
220015	Sleep-Wake Disorders	Insomnia Disorder
220016	Sleep-Wake Disorders	Insomnia Disorder
216011	Sleep-Wake Disorders	Insomnia Disorder
216012	Anxiety Disorders	Generalized Anxiety Disorder
216012	Sleep-Wake Disorders	Insomnia Disorder
216012	Obsessive-Compulsive and Related Disorders	Trichotillomania (Hair-Pulling Disorder)
216013	Neurodevelopmental Disorders	Specific Learning Disorder
216014	Depressive Disorders	not further specified
201016	Sleep-Wake Disorders	Insomnia Disorder (intermittent)
201016	Disruptive, Impulse-Control, and Conduct Disorders	Oppositional Defiant Disorder
220018	Sleep-Wake Disorders	Insomnia Disorder

220018	Disruptive, Impulse-Control, and Conduct Disorders	Oppositional Defiant Disorder
216016	Sleep-Wake Disorders	Insomnia Disorder
204014	Disruptive, Impulse-Control, and Conduct Disorders	Oppositional Defiant Disorder
204014	Neurodevelopmental Disorders	Specific Learning Disorder
204016	Disruptive, Impulse-Control, and Conduct Disorders	Oppositional Defiant Disorder
214022	Depressive Disorders	Unspecified Depressive Disorder
218004	Neurodevelopmental Disorders	Specific Learning Disorder
218004	Disruptive, Impulse-Control, and Conduct Disorders	Oppositional Defiant Disorder
202008	Disruptive, Impulse-Control, and Conduct Disorders	Oppositional Defiant Disorder
219019	Sleep-Wake Disorders	Insomnia Disorder
209008	(Not DSM)	Sensory Processing Disorder (mild)
203008	Sleep-Wake Disorders	Insomnia Disorder
218005	Anxiety Disorders	Generalized Anxiety Disorder
218005	Disruptive, Impulse-Control, and Conduct Disorders	Oppositional Defiant Disorder
218005	Sleep-Wake Disorders	Insomnia Disorder
203010	Neurodevelopmental Disorders	Specific Learning Disorder

No Stimulants Cohort

SubID	Comorbidity	
172164	Neurodevelopmental Disorders	Speech Sound Disorder
176357	Anxiety Disorders	not further specified
177815	Sleep-Wake Disorders	Insomnia Disorder
178454	Neurodevelopmental Disorders	Specific Learning Disorder
178741	Neurodevelopmental Disorders	Specific Learning Disorder
179374	Anxiety Disorders	not further specified
179566	Anxiety Disorders	not further specified
179566	Depressive Disorders	not further specified
179595	Disruptive, Impulse-Control, and Conduct Disorders	Oppositional Defiant Disorder
180061	Anxiety Disorders	not further specified
182414	Disruptive, Impulse-Control, and Conduct Disorders	Oppositional Defiant Disorder
184618	Anxiety Disorders	Generalized Anxiety Disorder
186110	Neurodevelopmental Disorders	Tic Disorders
187174	Neurodevelopmental Disorders	Specific Learning Disorder
189081	Trauma- and Stressor-Related Disorders	Posttraumatic Stress Disorder

Supplementary Table 2: Baseline demographic characteristics for different compliance levels and dropouts

	On Stimulants Cohort	No Stimulants Cohort	Total	**Playing >50% of recommended sessions**	**Playing <50% of recommended sessions**	**Dropouts**
**N**	130	76	206	163	43	27
**Age Mean (SD)**	10.6 (1.75)	10.5 (1.82)	10.6 (1.77)	10.4 (1.7)	11.2 (1.9)	10.5 (1.6)
**Sex (Male)**	98/130 (75.4%)	56/76 (73.7%)	154/206 (74.8%)	119/163 (73%)	35/43 (81.4%)	22/27 (81.5%)
**Race**						
White	106/130 (81.5%)	57/76 (75%)	163/206 (79.1%)	137/163 (84%)	26/43 (60.5%)	20/27 (74.1%)
Non-white/other	32/130 (24.6%)	24/76 (31.6%)	56/206 (27.2%)	37/163 (22.7%)	19/43 (44.2%)	7/27 (25.9%)
**Ethnicity**						
Hispanic or Latino	24/130 (18.5%)	20/76 (26.3%)	44/206 (21.4%)	39/163 (23.9%)	5/43 (11.6%)	6/27 (22.2%)

Not Hispanic or Latino	106/130 (81.5%)	54/76 (71.1%)	160/206 (77.7%)	123/163 (75.5%)	37/43 (86%)	21/27 (77.8%)

Note. Race allowed multiple selections, thus the number of answers in the white/non-white categories do not add up 100% of the number of children per cohort/group.
