# Supplementary material for: Effectiveness of a digital therapeutic as adjunct to treatment with medication in pediatric ADHD
Source: NPJ Digit Med. 2021 Mar 26;4:58. doi: 10.1038/s41746-021-00429-0 (PMC7997870; doi:10.1038/s41746-021-00429-0)
Supplement: Supplementary file 2 — Reporting Summary [file 41746_2021_429_MOESM2_ESM.pdf]

nature research

Corresponding author(s): Kollins

Last updated by author(s): Dec 23, 2020

Reporting Summary

Nature Research wishes to improve the reproducibility of the work that we publish. This form provides structure for consistency and transparency in reporting. For further information on Nature Research policies, see our Editorial Policies and the Editorial Policy Checklist.

Statistics

For all statistical analyses, confirm that the following items are present in the figure legend, table legend, main text, or Methods section.

n/a	Confirmed	
☐	☒	The exact sample size (n) for each experimental group/condition, given as a discrete number and unit of measurement
☐	☒	A statement on whether measurements were taken from distinct samples or whether the same sample was measured repeatedly
☐	☒	The statistical test(s) used AND whether they are one- or two-sided<br>*Only common tests should be described solely by name; describe more complex techniques in the Methods section.*
☐	☒	A description of all covariates tested
☐	☒	A description of any assumptions or corrections, such as tests of normality and adjustment for multiple comparisons
☐	☒	A full description of the statistical parameters including central tendency (e.g. means) or other basic estimates (e.g. regression coefficient) AND variation (e.g. standard deviation) or associated estimates of uncertainty (e.g. confidence intervals)
☒	☐	For null hypothesis testing, the test statistic (e.g. F, t, r) with confidence intervals, effect sizes, degrees of freedom and P value noted<br>*Give P values as exact values whenever suitable.*
☒	☐	For Bayesian analysis, information on the choice of priors and Markov chain Monte Carlo settings
☒	☐	For hierarchical and complex designs, identification of the appropriate level for tests and full reporting of outcomes
☐	☒	Estimates of effect sizes (e.g. Cohen's d, Pearson's r), indicating how they were calculated

Our web collection on statistics for biologists contains articles on many of the points above.

Software and code

Policy information about availability of computer code

Data collection	NA
Data analysis	SAS version 9.2 or higher

For manuscripts utilizing custom algorithms or software that are central to the research but not yet described in published literature, software must be made available to editors and reviewers. We strongly encourage code deposition in a community repository (e.g. GitHub). See the Nature Research guidelines for submitting code & software for further information.

Data

Policy information about availability of data

All manuscripts must include a data availability statement. This statement should provide the following information, where applicable:

- Accession codes, unique identifiers, or web links for publicly available datasets
- A list of figures that have associated raw data
- A description of any restrictions on data availability

The STARS-Adjunct Investigators agree to share de-identified individual participant data, the study protocol, and the statistical analysis plan with academic researchers 6 months after publication, and following completion of a Data Use Agreement. Proposals should be directed to medinfo@akiliinteractive.com.

Field-specific reporting

Please select the one below that is the best fit for your research. If you are not sure, read the appropriate sections before making your selection.

☒ Life sciences ☐ Behavioural & social sciences ☐ Ecological, evolutionary & environmental sciences

For a reference copy of the document with all sections, see nature.com/documents/nr-reporting-summary-flat.pdf

Life sciences study design

All studies must disclose on these points even when the disclosure is negative.

Sample size	Sample size estimation was performed and determined that an effective total of at least 66 non-medicated participants (No Stimulants cohort) would be needed to demonstrate an effect size of 0.40 with 90% power and 95% confidence. This target effect size was determined from our previous RCT study (STARS-ADHD), where the within group effect size on the primary (IRS) was 0.38. Since there was no prior data to estimate an effect size for the medicated participants (On Stimulants cohort), we hypothesized we might observe an attenuated effect (~25% decrease in effect size) and so this cohort was powered for a .30 effect size. This analysis revealed that at least 117 medicated participants (cohort 1) would be needed to demonstrate an effect size of 0.30 with 90% power at the 95% confidence level using a within-group t-test. Incorporating a 10% dropout rate required that at least 130 + 73 = 203 participants were planned to be enrolled in the study.
Data exclusions	No data were excluded. All analyses were conducted using a complete case analysis. In no situation were missing data to be imputed.
Replication	All methods were clearly and comprehensively described in the protocol and the pre-specified Statistical Analysis Plan (SAP).
Randomization	Participants were not randomly allocated to treatment condition. All participants in this study received the same treatment.
Blinding	Blinding was not relevant for this open-label study since all participants received the same treatment.

Reporting for specific materials, systems and methods

We require information from authors about some types of materials, experimental systems and methods used in many studies. Here, indicate whether each material, system or method listed is relevant to your study. If you are not sure if a list item applies to your research, read the appropriate section before selecting a response.

Materials & experimental systems

n/a	Involved in the study	
☒	☐	Antibodies
☒	☐	Eukaryotic cell lines
☒	☐	Palaeontology and archaeology
☒	☐	Animals and other organisms
☐	☒	Human research participants
☐	☒	Clinical data
☒	☐	Dual use research of concern

Methods

n/a	Involved in the study	
☒	☐	ChIP-seq
☒	☐	Flow cytometry
☒	☐	MRI-based neuroimaging

Human research participants

Policy information about studies involving human research participants

Population characteristics	Eligible patients were male/female children/adolescents aged 8-14 years old with a confirmed diagnosis of Attention Deficit Hyperactivity Disorder (as per the Diagnostic and Statistical Manual of Mental Disorders (5th edn)), primarily inattentive or combined subtype (per DSM-V and MINI-KID), experiencing suboptimal treatment response, as reflected by ADHD-related impairment (IRS Overall Impairment Score ≥ 3, parent-rated) at screening, and no significant comorbid psychiatric diagnoses that would make participation in the study difficult. All participants had an IQ score ≥ 80 (per KBIT-II).<br>For the On Stimulants cohort, participants had to be on a stable dose of stimulant medication, at an approved dose, for at least 30 days prior to enrollment and show a moderate response on a stimulant, with room for improvement. For the No Stimulants cohort, participants had to be stable without a stimulant or any other ADHD medication for at least 30 days before the baseline visit.
Recruitment	Eligible participants were recruited by a variety of methods at each of the study sites, including advertising, and review of existing patient records. Sites represented a range of geographic and urban/surburban settings and attention was given to ensuring a diverse sample so as to minimize the effects of self-selection. Sample characteristics (demographics) were comparable to the population from which it was drawn. Participation was open to any patient who met eligibility criteria, but it is possible that some degree of self-selection took place. The generalizability of the findings is discussed in detail in the Discussion section.

Ethics oversight	The study was conducted in accordance with the International Conference on Harmonisation Regulations, and was approved by each site's institutional review board (Copernicus Group/WIRB [11 sites], Cincinnati Children's Hospital Medical Center, UC Davis, USCF, Johns Hopkins Medical Center [1 site each]. The overall study IRB was Duke University Health System). All participants and their caregivers provided written or verbal assent and written consent, respectively, prior to any study activities being conducted.

Note that full information on the approval of the study protocol must also be provided in the manuscript.

Clinical data

Policy information about clinical studies

All manuscripts should comply with the ICMJE guidelines for publication of clinical research and a completed CONSORT checklist must be included with all submissions.

Clinical trial registration	NCT03649074
Study protocol	*Note where the full trial protocol can be accessed OR if not available, explain why.*
Data collection	*Describe the settings and locales of data collection, noting the time periods of recruitment and data collection.*
Outcomes	*Describe how you pre-defined primary and secondary outcome measures and how you assessed these measures.*
